# Supplementary material for: Molecular Markers of Telomerase Complex for Patients with Pituitary Adenoma
Source: Brain Sci. 2022 Jul 25;12(8):980. doi: 10.3390/brainsci12080980 (PMC9331889; doi:10.3390/brainsci12080980)
Supplement: Supplementary file 1 [file brainsci-12-00980-s001.zip › brainsci-1752057-supplementary.pdf]

Supplementary Material

Table S1. Genotype and allele frequencies of single nucleotide polymorphisms (*TEP1* rs1760904, rs1713418, *TERC* rs12696304, *TERT* rs2736098, rs401681) within PA and control groups.

Gene, SNP	Genotype, allele	PA group, N (%)	Control group, N (%)	p-value
TEP1 rs1760904	AA	33 (26.2)	95 (25.8)	0.845
	AG	66 (52.4)	185 (50.3)	
	GG	27 (21.4)	88 (23.9)	
	Total	126 (100)	368 (100)	
	Allele			
	A	132 (52.4)	375 (51.0)	0.695
	G	120 (47.6)	361 (49.0)	
TEP1 rs1713418	AA	45 (35.7)	136 (37.0)	0.120
	AG	69 (54.8)	172 (46.7)	
	GG	12 (9.5)	60 (16.3)	
	Total	126 (100)	368 (100)	
	Allele			
	A	159 (63.1)	444 (60.3)	0.437
	G	93 (36.9)	292 (39.7)	
TERC rs12696304	CC	64 (50.8)	199 (54.1)	0.687
	CG	54 (42.9)	142 (38.6)	
	GG	8 (6.3)	27 (7.3)	
	Total	126 (100)	368 (100)	
	Allele			
	A	182 (72.2)	540 (73.4)	0.723
	G	70 (27.8)	196 (26.6)	
TERT rs2736098	CC	72 (57.1)	221 (60.1)	0.223
	CT	38 (30.2)	119 (32.3)	
	TT	16 (12.7)	28 (7.6)	
	Total	126 (100)	368 (100)	
	Allele			
	C	182 (72.2)	561 (76.2)	0.204
	T	70 (27.8)	175 (23.8)	
TERT rs401681	CC	47 (37.3)	126 (34.2)	0.732
	CT	60 (47.6)	177 (48.1)	
	TT	19 (15.1)	65 (17.7)	
	Total	126 (100)	368 (100)	
	Allele			
	C	143 (56.7)	429 (58.3)	0.075
	T	77 (43.3)	307 (41.7)	

Table S2. *TEP1*, *TERC*, and *TERT* genes single nucleotide polymorphisms frequencies of genotypes and alleles within active or inactive pituitary adenoma and control groups.

Gene, SNP	Genotype, allele	Control group, N (%)	Active PA group, N (%)	p-value	Inactive PA group, N (%)	p-value
TEP1 rs1760904	AA	95 (25.8)	17 (25.4)	0.997	15 (27.3)	0.815
	AG	185 (50.3)	34 (50.7)		29 (52.7)	
	GG	88 (23.9)	16 (23.9)		11 (20.0)	
	Total	368 (100)	67 (100)		55 (100)	
	Allele					
	A	375 (51.0)	68 (40.7)	0.960	59 (53.6)	0.672
	G	361 (49.0)	66 (49.3)		51 (46.4)	
TERC rs12696304	CC	199 (54.1)	31 (46.3)	0.687	30 (54.5)	0.581
	CG	142 (38.6)	30 (44.8)		23 (41.8)	
	GG	27 (7.3)	6 (9.0)		2 (3.6)	
	Total	368 (100)	67 (100)		55 (100)	
	Allele					
	A	540 (73.4)	92 (68.7)	0.308	83 (75.5)	0.729
	G	196 (26.6)	42 (31.3)		27 (24.5)	
TERT rs2736098	CC	221 (60.1)	33 (49.3)	0.209	35 (63.6)	0.105
	CT	119 (32.3)	26 (38.8)		12 (21.8)	
	TT	28 (7.6)	8 (11.9)		8 (14.5)	
	Total	368 (100)	67 (100)		55 (100)	
	Allele					
	C	561 (76.2)	92 (68.7)	0.080	82 (74.5)	0.791
	T	175 (23.8)	42 (31.3)		28 (25.5)	
TERT rs401681	CC	126 (34.2)	24 (35.8)	0.859	23 (41.8)	0.542
	CT	177 (48.1)	33 (49.3)		23 (41.8)	
	TT	65 (17.7)	10 (14.9)		9 (16.4)	
	Total	368 (100)	67 (100)		55 (100)	
	Allele					
	C	429 (58.3)	81 (60.4)	0.710	69 (62.7)	0.436
	T	307 (41.7)	53 (39.6)		41 (37.3)	

Table S3. *TEP1*, *TERC*, and *TERT* genes single nucleotide polymorphisms frequencies of genotypes and alleles within invasive or non-invasive pituitary adenoma and control groups.

Gene, SNP	Genotype, allele	Control group, N (%)	Invasive PA group, N (%)	p-value	Non-invasive PA group, N (%)	p-value
TEP1 rs1760904	AA	95 (25.8)	24 (29.6)	0.538	8 (18.2)	0.538
	AG	185 (50.3)	42 (51.9)		24 (54.5)	
	GG	88 (23.9)	15 (18.5)		12 (27.3)	
	Total	368 (100)	81 (100)		44 (100)	
	Allele					
	A	375 (51.0)	90 (55.6)	0.288	40 (45.5)	0.330
	G	361 (49.0)	72 (44.4)		48 (54.5)	
TERC rs12696304	CC	199 (54.1)	43 (53.1)	0.431	20 (45.5)	0.453
	CG	142 (38.6)	35 (43.2)		19 (43.2)	
	GG	27 (7.3)	3 (3.7)		5 (11.4)	
	Total	368 (100)	81 (100)		44 (100)	
	Allele					
	A	540 (73.4)	121 (74.7)	0.730	59 (67.0)	0.208
	G	196 (26.6)	41 (25.3)		29 (33.0)	
TERT rs401681	CC	126 (34.2)	33 (40.7)	0.524	14 (31.8)	0.870
	CT	177 (48.1)	36 (44.4)		23 (52.3)	
	TT	65 (17.7)	12 (14.8)		7 (15.9)	
	Total	368 (100)	81 (100)		44 (100)	
	Allele					
	C	429 (58.3)	102 (63.0)	0.273	51 (58.0)	0.952
	T	307 (41.7)	60 (37.0)		37 (42.0)	

Table S4. *TEP1*, *TERC*, and *TERT* genes single nucleotide polymorphisms frequencies of genotypes and alleles within pituitary adenoma with relapse or without relapse and control groups.

Gene, SNP	Genotype, allele	Control group, N (%)	PA group with relapse, N (%)	p-value	PA group without relapse, N (%)	p-value
TEP1 rs1760904	AA	95 (25.8)	8 (26.7)	0.393	24 (26.1)	0.968
	AG	185 (50.3)	18 (60.0)		45 (48.9)	
	GG	88 (23.9)	4 (13.3)		23 (25.0)	
	Total	368 (100)	30 (100)		92 (100)	
	Allele					
	A	375 (51.0)	34 (45.7)	0.373	93 (50.5)	0.921
	G	361 (49.0)	26 (43.3)		91 (49.5)	
TERC rs12696304	CC	199 (54.1)	14 (46.7)	0.683	47 (51.1)	0.794
	CG	142 (38.6)	14 (46.7)		39 (42.4)	
	GG	27 (7.3)	2 (6.6)		6 (6.5)	
	Total	368 (100)	30 (100)		92 (100)	
	Allele					
	A	540 (73.4)	42 (70.0)	0.571	133 (72.3)	0.766
	G	196 (26.6)	18 (30.0)		51 (27.7)	
TERT rs401681	CC	126 (34.2)	9 (30.0)	0.881	38 (41.3)	0.414
	CT	177 (48.1)	15 (50.0)		41 (44.6)	
	TT	65 (17.7)	6 (20.0)		13 (14.1)	
	Total	368 (100)	30 (100)		92 (100)	
	Allele					
	C	429 (58.3)	33 (55.0)	0.620	117 (63.6)	0.191
	T	307 (41.7)	27 (45.0)		67 (36.4)	

Table S5. *TEP1*, *TERC*, and *TERT* genes single nucleotide polymorphisms frequencies of genotypes and alleles within macro or micro pituitary adenoma and control groups.

Gene, SNP	Genotype, allele	Control group, N (%)	Macro PA, N (%)	p-value	Micro PA, N (%)	p-value
TEP1 rs1760904	AA	95 (25.8)	20 (25.3)	0.966	12 (29.3)	0.407
	AG	185 (50.3)	39 (49.4)		23 (56.1)	
	GG	88 (23.9)	20 (25.3)		6 (14.6)	
	Total	368 (100)	79 (100)		41 (100)	
	Allele					
	A	375 (51.0)	79 (50.0)	0.828	47 (57.3)	0.274
	G	361 (49.0)	79 (50.0)		35 (42.7)	
TEP1 rs1713418	AA	136 (37.0)	28 (35.4)	0.445	17 (41.5)	0.153
	AG	172 (46.7)	42 (53.2)		22 (53.7)	
	GG	60 (16.3)	9 (11.4)		2 (4.8)	
	Total	368 (100)	79 (100)		41 (100)	
	Allele					
	A	444 (60.3)	98 (62.0)	0.692	56 (68.3)	0.160
	G	292 (39.7)	60 (38.0)		26 (31.7)	
TERC rs12696304	CC	199 (54.1)	36 (45.6)	0.197	23 (56.1)	0.778
	CG	142 (38.6)	39 (49.4)		14 (34.1)	
	GG	27 (7.3)	4 (5.1)		4 (9.8)	
	Total	368 (100)	79 (100)		41 (100)	
	Allele					
	A	540 (73.4)	111 (70.3)	0.424	60 (73.2)	0.969
	G	196 (26.6)	47 (29.7)		22 (26.8)	
TERT rs401681	CC	126 (34.2)	30 (38.0)	0.672	17 (41.5)	0.638
	CT	177 (48.1)	38 (48.1)		17 (41.5)	
	TT	65 (17.7)	11 (13.9)		7 (17.0)	
	Total	368 (100)	79 (100)		41 (100)	
	Allele					
	C	429 (58.3)	98 (62.0)	0.386	51 (62.2)	0.496
	T	307 (41.7)	60 (38.0)		31 (37.8)	

Table S6. Frequencies of genotypes and serum TEP1 levels.

Genotype	Serum TEP1 level		P-value*
	PA group median (IQR)	Control group median (IQR)	
TEP1 rs1760904			
AA	240.5 (111)	272.18 (180.12)	0.366
AG+GG	235.83 (173.86)	268.89 (199.71)	0.616
TERC rs12696304			
CC	254.30 (141.52)	269.26 (193.06)	0.464
CG+GG	228.80 (146.63)	234.51 (132.14)	1.000
TERC rs35073794			
GG	-	258.09 (165.74)	N/A
AG+AA	235.83 (127.19)	326.76 (189.41)	0.196
TERT rs2736098			
CC	254.30 (406.35)	282.99 (251.93)	0.891
CT+TT	228.80 (129.18)	268.89 (153.22)	0.308
TERT rs401681			
CC	272.76 (87.16)	261.42 (146.86)	0.866
CT+TT	231.07 (146.08)	268.89(197.58)	0.242

* Mann-Whitney U test

Table S7. Frequencies of genotypes and relative leukocyte telomeres length.

Genotype	Relative leukocyte telomeres length		P-value
	PA group Median (IQR)	Control group Median (IQR)	
TEP1 rs1760904			
AA	0.198 (1.05)	0.511 (0.84)	0.172*
AG+GG	0.624 (0.54)	0.822 (1.31)	0.360*
TEP1 rs1713418			
AA	0.331 (1.07)	0.613 (0.73)	0.372*
AG+GG	0.857 (1.13)	0.603 (0.55)	0.525*
TERC rs12696304			
CC	0.361 (0.89)	0.539 (0.66)	0.301*
CG+GG	0.932 (1.57)	0.633 (0.58)	0.199**
TERC rs35073794			
GG	-	0.631 (0.90)	N/A
AG+AA	0.645 (1.11)	0.576 (0.43)	0.774*
TERT rs2736098			
CC	0.440 (1.26)	0.561 (0.59)	0.561*
CT+TT	0.944 (1.04)	0.665 (0.67)	0.645*
TERT rs401681			
CC	1.166 (1.88)	0.661 (0.68)	0.137*
CT+TT	0.411 (0.85)	0.596 (0.55)	0.204*

* Mann-Whitney U test was used; ** Stjudent T test was used

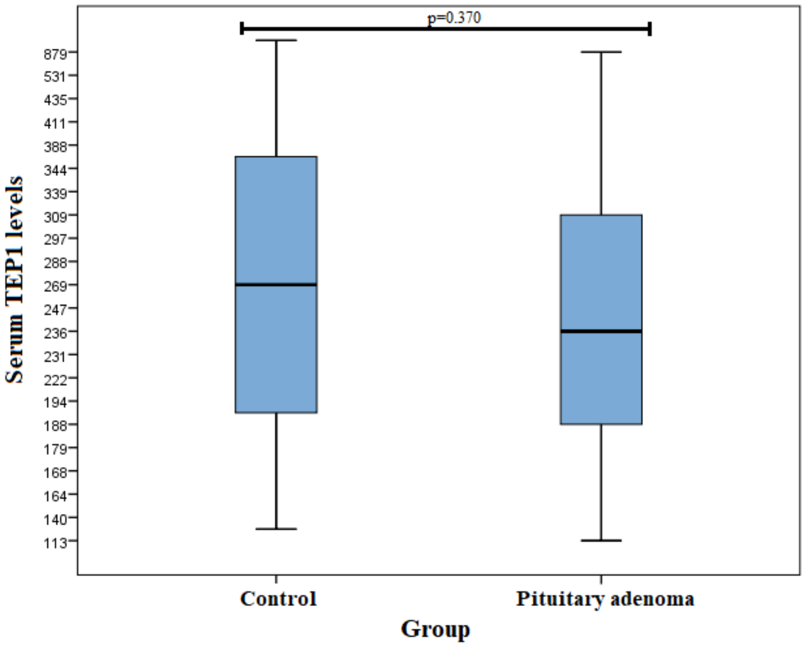

Figure S1. Serum TEP1 levels in PA and control groups.

Mann-Whitney U test was used.

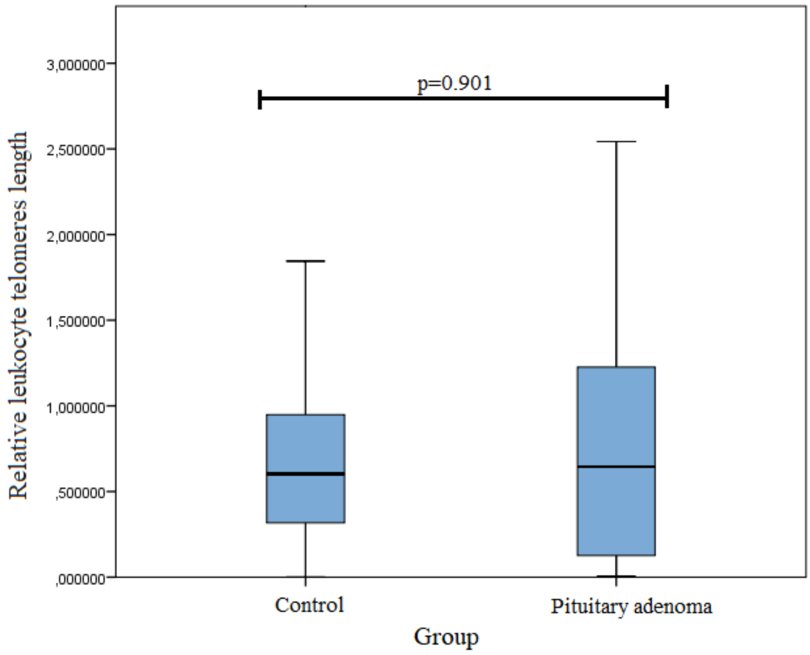

Figure S2. Relative leukocyte telomeres length between PA and control groups.

Mann-Whitney U test was used
